# Supplementary material for: Feasibility of Using a Risk Assessment Tool to Predict Hospital Transfers or Death for Older People in Australian Residential Aged Care. A Retrospective Cohort Study
Source: Healthcare (Basel). 2020 Aug 21;8(3):284. doi: 10.3390/healthcare8030284 (PMC7551645; doi:10.3390/healthcare8030284)
Supplement: Supplementary file 1 [file healthcare-08-00284-s001.pdf]

healthcare MDPI

Supplement 1. Modified CriSTAL tool for Residential Aged Care

Completion date / / Participant Study ID ________________

Date of RACF admission as permanent resident: / /

	**Sex 1. □ Male 2. □ Female**<br>**Date of birth ______/_______ /_____ or age if DOB not available**
☐	**Age > 60** (1 point)
☐	RACF permanent resident or admitted permanently during the study period (1 point)
☐	**Frailty indicators** ☐ Yes ☐ No Based on activities of daily living (specify)<br>____<br>Estimated Rockwood score >= 5 (1 point if yes) (Estimated Clinical Frailty Score 1-9)<br>_________
**AND**	OTHER RISK FACTORS /PREDICTORS (*Tick as many as relevant*) (1 point for each condition)
	**Personal history of active disease:**
☐	1-Advanced malignancy
☐	2-Chronic kidney disease
☐	3-Chronic heart failure
☐	4-Chronic obstructive pulmonary disease
☐	5-New cerebrovascular disease
☐	6-New myocardial infarction
	7-Moderate/severe liver disease
☐	Evidence of cognitive impairment (mark as many as relevant) (1 point if at least 1 condition)<br>☐ Dementia ☐ Long term mental disorder (depression or anxiety)<br>☐ Behavioural Alterations ☐ Mental disability from stroke
☐	Nutritional vulnerability on admission (1 point if at least 1 condition)<br>☐Malnutrition, ☐Sarcopenia ☐History of unintentional weight loss<br>☐Feeding dependency ☐Feeding tube
☐	History of pneumonia in the 6 months leading to RACF admission (1 point if yes)
☐	Fall in the past 3 months (before becoming a resident) (1 point if yes)<br>☐ Yes ☐ No ☐ Not documented
☐	Polypharmacy (**7 or more** medications) on admission to RACF (1 point if yes)<br>Total number of medications _________
☐	**Previous hospitalisation for at least one night in past year** (1 point if >1 hospital admission)

☐	☐ Yes ☐ No ☐ Not documented<br>**ICU admission at previous hospitalisation in the past year** (1 point if >1 ICU admission)<br>☐ Yes ☐ No ICU admission at all ☐ Unknown

CriSTAL score ___________

Clinical Frailty Scale

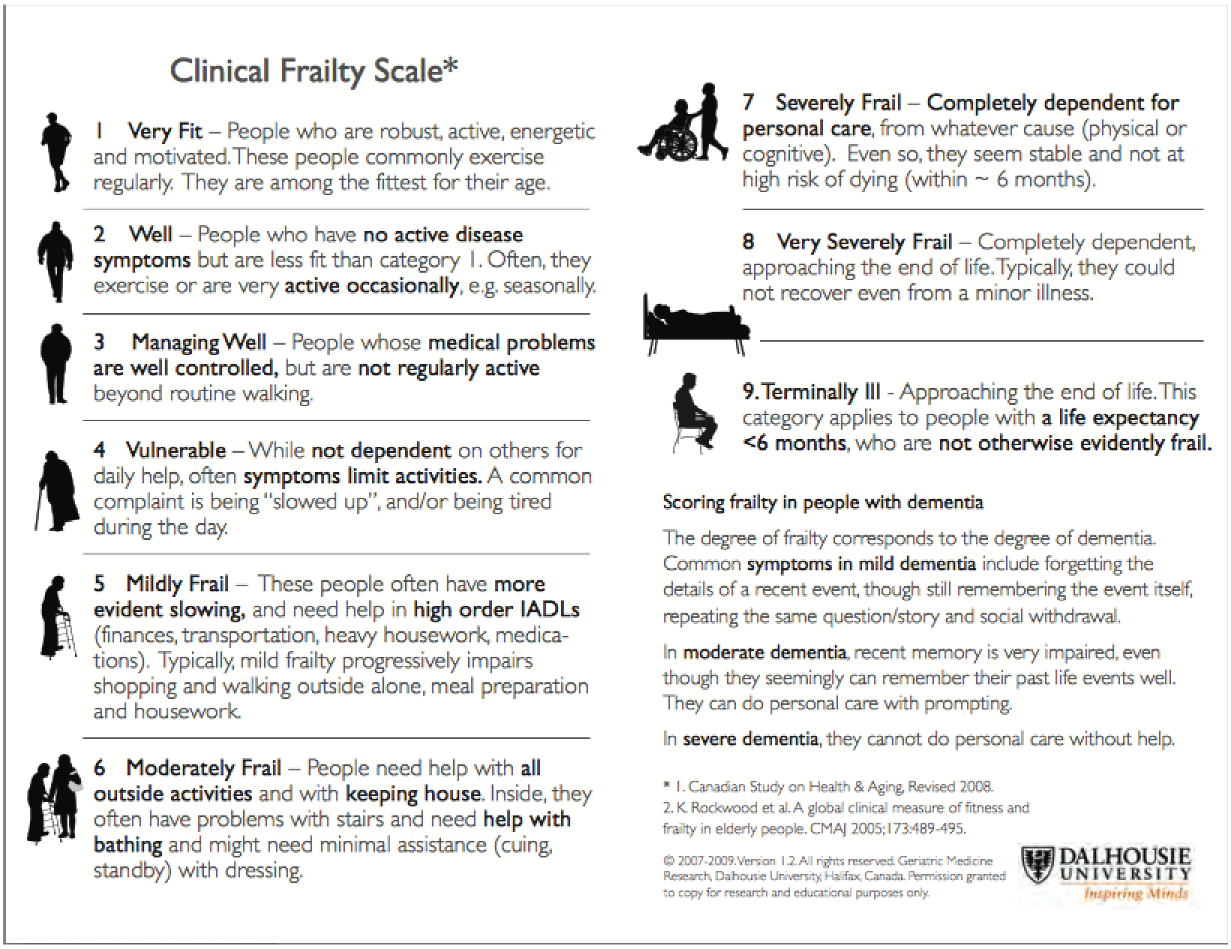

Clinical Frailty Scale*

1 **Very Fit** – People who are robust, active, energetic and motivated. These people commonly exercise regularly. They are among the fittest for their age.

2 **Well** – People who have **no active disease symptoms** but are less fit than category 1. Often, they exercise or are very **active occasionally**, e.g. seasonally.

3 **Managing Well** – People whose **medical problems are well controlled,** but are **not regularly active** beyond routine walking.

4 **Vulnerable** – While **not dependent** on others for daily help, often **symptoms limit activities.** A common complaint is being "slowed up", and/or being tired during the day.

5 **Mildly Frail** – These people often have **more evident slowing,** and need help in **high order IADLs** (finances, transportation, heavy housework, medications). Typically, mild frailty progressively impairs shopping and walking outside alone, meal preparation and housework.

6 **Moderately Frail** – People need help with **all outside activities** and with **keeping house.** Inside, they often have problems with stairs and need **help with bathing** and might need minimal assistance (cuing, standby) with dressing.

7 **Severely Frail** – **Completely dependent for personal care,** from whatever cause (physical or cognitive). Even so, they seem stable and not at high risk of dying (within ~ 6 months).

8 **Very Severely Frail** – Completely dependent, approaching the end of life. Typically, they could not recover even from a minor illness.

9. **Terminally Ill** - Approaching the end of life. This category applies to people with **a life expectancy <6 months,** who are **not otherwise evidently frail.**

Scoring frailty in people with dementia

The degree of frailty corresponds to the degree of dementia. Common **symptoms in mild dementia** include forgetting the details of a recent event, though still remembering the event itself, repeating the same question/story and social withdrawal.

In **moderate dementia,** recent memory is very impaired, even though they seemingly can remember their past life events well. They can do personal care with prompting.

In **severe dementia,** they cannot do personal care without help.

* 1. Canadian Study on Health & Aging, Revised 2008.
2. K. Rockwood et al. A global clinical measure of fitness and frailty in elderly people. CMAJ 2005;173:489-495.

© 2007-2009. Version 1.2. All rights reserved. Geriatric Medicine Research, Dalhousie University, Halifax, Canada. Permission granted to copy for research and educational purposes only.

DALHOUSIE UNIVERSITY *Inspiring Minds*

Rockwood K, Song X, MacKnight C, et al. A global clinical measure of fitness and frailty in elderly people. *CMAJ: Canadian Medical Association journal = journal de l'Association medicale canadienne* 2005;173(5):489-95. doi: 10.1503/cmaj.050051
